# Evaluation of the Ebola Virus Disease (EVD) preparedness and readiness program in Uganda: 2018 to 2019

**DOI:** 10.11604/pamj.2021.38.130.27391

**Published:** 2021-02-04

**Authors:** Peter Nsubuga, Ben Masiira, Christine Kihembo, Jayne Byakika-Tusiime, Caroline Ryan, Miriam Nanyunja, Raoul Kamadjeu, Ambrose Talisuna

**Affiliations:** 1 Global Public Health Solutions, Atlanta Georgia, United States of America,; 2 Epidemiologist, Kampala, Uganda,; 3 World Health Organization, Uganda Country Office, Kampala, Uganda,; 4 World Health Organization, Sub-Regional Office, Nairobi, Kenya,; 5 Epidemiologist, New York, United States of America,; 6 World Health Organization, Regional Office for Africa, Brazzaville, Congo

**Keywords:** Ebola virus disease, evaluation, Uganda

## Abstract

**Introduction:**

the Democratic Republic of Congo (DRC) declared its 10^th^outbreak of Ebola virus disease (EVD) in 42 years on August 1^st^ 2018. The rapid rise and spread of the EVD outbreak threatened health security in neighboring countries and global health security. The United Nations developed an EVD preparedness and readiness (EVD-PR) plan to assist the nine neighboring countries to advance their critical preparedness measures. In Uganda, EVD-PR was implemented between 2018 and 2019. The World Health Organization commissioned an independent evaluation to assess the impact of the investment in EVD-PR in Uganda. Objectives: i) to document the program achievements; ii) to determine if the capacities developed represented good value for the funds and resources invested; iii) to assess if more cost-effective or sustainable alternative approaches were available; iv) to explore if the investments were aligned with country public health priorities; and v) to document the factors that contributed to the program success or failure.

**Methods:**

during the EVD preparedness phase, Uganda's government conducted a risk assessment and divided the districts into three categories, based on the potential risk of EVD. Category I included districts that shared a border with the DRC provinces where EVD was ongoing or any other district with a direct transport route to the DRC. Category II were districts that shared a border with the DRC but not bordering the DRC provinces affected by the EVD outbreak. Category III was the remaining districts in Uganda. EVD-PR was implemented at the national level and in 22 category I districts. We interviewed key informants involved in program design, planning and implementation or monitoring at the national level and in five purposively selected category I districts.

**Results:**

Ebola virus disease preparedness and readiness was a success and this was attributed mainly to donor support, the ministry of health's technical capacity, good coordination, government support and community involvement. The resources invested in EVD-PR represented good value for the funds and the activities were well aligned to the public health priorities for Uganda.

**Conclusion:**

Ebola virus disease preparedness and readiness program in Uganda developed capacities that played an essential role in preventing cross border spread of EVD from the affected provinces in the DRC and enabled rapid containment of the two importation events. These capacities are now being used to detect and respond to the COVID-19 pandemic.

## Introduction

Ebola virus disease (EVD) remains a severe public health challenge in Africa [1]. The Democratic Republic of Congo (DRC) declared its 10^th^ outbreak of Ebola virus disease (EVD) in 42 years on August 1^st^, 2018 [2] which was centered in the east of the country and it declared the 11^th^ outbreak on June 1^st^ 2020 [3]. The 10^th^ EVD outbreak in the DRC was the country's largest-ever Ebola outbreak and it was the second largest Ebola epidemic recorded, after the West Africa outbreak of 2013 to 2016. By the end of the outbreak in June 2020, a total of 3,463 EVD cases, including 3,317 confirmed and 146 probable cases had been reported of which 2,280 patients died (overall case fatality ratio 66%) and 1,171 had recovered [4].

Despite the DRC's commendable efforts to contain the outbreak, the risk of spread to other areas within and beyond the DRC borders was exceedingly high. It included two incidences where confirmed cases crossed to Uganda in June and August 2019 [5,6]. The first confirmed cases that crossed the border to Uganda were three family members from the DRC. They subsequently died from EVD with no further transmission or secondary cases within Uganda. There were significant challenges to breaking the chain of transmission and ending this outbreak in the DRC. Unlike other areas in the DRC where Ebola had been successfully contained, the 10^th^ EVD outbreak occurred in an extremely fragile environment marked by an active and prolonged civil conflict and the presence of several armed groups [7]. The concurrence of several other outbreaks with similar non-specific clinical signs such as measles and malaria also challenged the initial clinical diagnosis [8]. Specifically, the spread of the Ebola zaire virus in cholera-affected health zones in the DRC (i.e. Kayna and Alimbongo) in 2019 complicated response activities [9].

The World Health Organization (WHO) declared the Ebola outbreak in the DRC, a public health emergency of international concern (PHEIC) on July 17^th^ 2019 [10]. WHO recommended that the at-risk neighboring countries implement several activities to strengthen their EVD preparedness. To assist the DRC's nine neighboring countries with advancing critical preparedness measures, the United Nations developed a plan entitled “regional Ebola preparedness: overview of needs and requirements July - December 2019” [11]. The regional plan served as a complement to the integrated strategy to respond to Ebola virus: Ituri and North Kivu provinces for the DRC covering the same period [12]. Together, the two documents presented the full scope of actions and funding required to respond to the EVD outbreak in the DRC, prevent it from spreading further and drive the response towards zero cases. The nine countries included in the regional overview were categorized into two groups, based on risk level: priority 1 (based on proximity to areas where cases were reported and where the large-scale movement of goods and people across borders was occurring): Burundi, Rwanda, South Sudan and Uganda; priority 2 (all other countries neighboring the DRC): Angola, Central African Republic, Republic of Congo, Tanzania and Zambia.

The regional plan presented a consolidated summary of urgent activities required to advance preparedness, as elaborated in each country's national strategy, focusing on priority 1 countries. At the end of the regional plan, WHO commissioned an independent evaluation to determine the impact of the investment in preparedness and readiness in some of the countries neighboring the DRC. The evaluation's objectives were to document achievements during the EVD preparedness period and determine which could be attributed to the investments in enhancing EVD preparedness; determine if the capacities developed for EVD preparedness and readiness represented good value for the funds and resources invested in the country. Other objectives were to explore if more cost-effective or sustainable alternative approaches exist in the country that could have delivered similar results and if the investments were aligned with the priority needs to improve health system security, and in line with country international health regulations (IHR) (2005) obligations. The last objective was to understand and document other factors contributing to the interventions' success or failure. Uganda has had considerable familiarity with EVD, having experienced six outbreaks since 2000 [13]. This paper describes the process and the results of the independent evaluation of the EVD preparedness program conducted in Uganda between May and September 2020.

## Methods

Between May and September 2020, based on the WHO evaluation framework [14], we used the objectives of the evaluation to develop a set of evaluation questions which were nested in an evaluation design matrix. We developed a set of evaluation tools to obtain information that would enable us to answer the evaluation questions. The evaluation questions were as follows: 1) what were the achievements during EVD preparedness period and how are these attributed to the investments made to enhance EVD preparedness; 2) do the capacities developed for EVD preparedness and readiness represent good value for the funds and resources invested in the country; 3) were there more cost-effective or sustainable alternative approaches that could have delivered similar or improved results towards strengthening EVD preparedness capacity; 4) are the investments into EVD preparedness and readiness aligned with the country's priority needs to strengthen health system security for public health emergencies and align with their IHR (2005) obligations; 5) what other factors contributed to the success or failure of the interventions?

**Figure 1:**
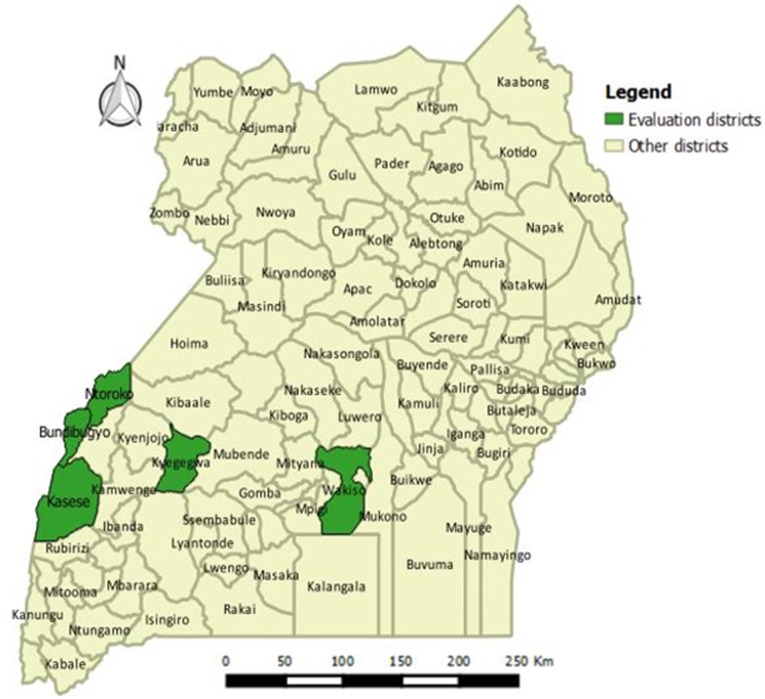
a map highlighting the five evaluation districts EVD preparedness and readiness evaluation, Uganda 2020

We evaluated five districts in Uganda: Bundibugyo, Kasese, Kyegegwa, Ntoroko and Wakiso during June and July 2020 (Figure 1). We also obtained information from national-level staff and development partners. When the EVD outbreak was declared in Eastern DRC on August 1^st^ 2018, the Uganda government collaborated with technical and development partners and developed a national preparedness and response plan, which divided the districts into three categories. Category I included 20 districts that shared a common border with EVD affected provinces in the DRC where the EVD outbreak was active. Category II included districts that shared a border with the DRC but not directly bordering EVD affected provinces. Category III included the remaining districts in Uganda.

**Evaluation design and selection of evaluation districts:** the evaluation was conducted in category I districts described above. During the program's implementation, some category I districts were prioritized to receive more interventions based on population movements between the two countries and the availability of funding. The five districts where the evaluation was implemented were purposively selected because they received the full package of the targeted interventions for EVD preparedness.

**Selection of evaluation participants:** the key informants were technical officers selected at national and district levels. The key informants were purposively selected based on their involvement in designing, planning, implementation and monitoring the EVD preparedness and readiness program. The national-level key informants included the EVD Incident manager, emergency operations center (EOC) manager, epidemiology and surveillance department (ESD), Head who was the EVD Incident manager, heads of response sub-committees, ministry of health senior epidemiologists involved in EVD preparedness and response and senior managers within development partner technical agencies supporting the ministry of health. The participants included the district health officer, district laboratory focal person, district surveillance officer and the district biostatistician at the district level.

**Data collection:** we collected quantitative and qualitative information from participants at the national and sub-national levels. For the quantitative data, an online survey questionnaire was sent out to 40 individuals, of which 27 (67.5%) responded. The data collected included opinions on the program achievements, value for money, availability of more cost-effective alternative interventions, alignment of the program with national public health priorities and factors contributing to the interventions' success. A Likert scale was used to assess key informants' opinions about the different aspects of the EVD preparedness and readiness program. We collected qualitative data from 32 key Informants using face-to-face interviews to complement the quantitative data's interpretation and explore more opinions about other program-related issues.

**Data analysis:** we performed a mixed analysis comprising quantitative analysis and qualitative methods. Quantitative variables were grouped into different categories and frequencies were generated and tabulated using Microsoft Excel and Epi Info version 7. We conducted a thematic analysis for qualitative information by reviewing the interview transcripts to identify common themes from the responses.

**Ethical issues:** this was an evaluation of a public health program which was determined to be non-human subject research according to Uganda's research guidelines [15]. The ministry of health national task force on epidemics and public health emergencies approved the evaluation protocol. The potential evaluation participants were provided with information about the evaluation through e-mail and face-to-face interviews. They were assured that their participation was voluntary and their refusal would not lead to any consequences. For the face-to-face interviews, we obtained verbal consent from each participant before the interview.

**Ethics approval and consent:** the evaluation was requested by the Uganda Ministry of Health and the national task force on epidemics and public health emergencies approved the evaluation protocol. We interviewed participants only after obtaining verbal consent. The MOH national task force approved the consent procedures on epidemics and public health emergencies.

## Results

**Description of respondents:** we targeted 40 key informants for the online questionnaire (quantitative) survey, 27 (67.5%) responded of which 70% were national-level officers and 30% were district level officers (Table 1). We conducted qualitative interviews with 32 (91%) of the 35 we targeted (Table 1).

**Table 1:** characteristics of respondents in the EVD preparedness and readiness evaluation, Uganda 2020

Characteristic	Quantitative assessment		Qualitative assessment	
	Number	Percent	Number	Percent
**Response rate**				
Targeted number	40	100	35	100
Number interviewed	27	67.5	32	91.4
Duty station				
National level	19	70.3	11	34.4
Sub-national level	8	29.7	21	65.6

**The success of the EVD preparedness and readiness program:** the online survey respondents were asked to provide a broad opinion about the program's success; 25 (92.6%) out of 27 reported that the entire program was successful. Most of the respondents agreed that the response pillars-sub-committees were more prepared to respond to EVD outbreaks than the period before the program was implemented. The levels of agreement by pillar were; surveillance and laboratory - 96.3% (26/27), risk communication, 96.0% (24/25), case management and IPC - 88.9% (24/27), coordination - 88.5% (23/26), logistics - 87.5% (21/24) and psycho-social support - 79.2% (19/24) (Table 2). Results from qualitative key informant interviews supported the quantitative findings. The benefits attributed to the program were analyzed under eight broad category key themes which included: i) knowledge and skills gain; ii) strengthening public health surveillance; iii) improved cross-border collaboration; ix) improved infection prevention and control; x) improved coordination for preparedness and response; xi) community empowerment; and xii) supply and logistical support (Table 3).

**Table 2:** opinions of key informants about the EVD preparedness and readiness program respondents in the EVD preparedness and readiness evaluation, Uganda 2020

Element	Agree		Disagree		Not sure		Total
	n	%	n	%	n	%	
The entire program was a success	25	92.6%	1	3.7%	1	3.7%	27
The coordination pillar is now more prepared to respond to EVD outbreaks	23	88.5%	1	3.8%	0	0%	26
The surveillance and laboratory pillar is now more prepared to respond to EVD outbreaks	26	96.3%	1	3.7%	0	0%	27
The case management and IPC pillar is now more prepared to respond to EVD outbreaks	24	88.9%	1	3.7%	1	3.7%	27
The risk communication pillar is now more prepared to respond to EVD outbreaks	24	96.0%	1	4.5%	0	0%	25
The psychosocial support pillar is now more prepared to respond to EVD outbreaks	19	79.2%	5	20.8%	0	0%	24
The logistics pillar is now more prepared to respond to EVD outbreaks	21	87.5%	1	4.2%	2	8.3%	24
The program had value for money	25	92.6%	1	3.7%	1	3.7%	27
There were more cost-effective alternative interventions to deliver similar results	9	34.6%	6	23.1%	11	42.3%	26
The investments were aligned to the national public health priorities	22	84.6%	1	3.8%	2	7.7%	26

**Table 3:** opinions of key informants about the main achievements of the program respondents in the EVD preparedness and readiness evaluation, Uganda 2020

No.	Theme	Main issues/achievements reported (n=32)
1.0	Knowledge and skills gain	Health workers gained knowledge
		The program empowered health workers with skills to investigate VHFs and other outbreaks
		Health workers feel more comfortable to respond to EVD without fear
		National task force (NTF) and district task forces were trained on coordination
		The VHTs received training on epidemics
		Knowledge acquisition through simulation exercises that were conducted
2.0	Strengthening public health surveillance	Improved epidemic/case detection
		Reporting for IDSR priority diseases has improved
		WHO deployed technical officers and consultants to support surveillance, IPC and other activities
		Laboratory capacities were strengthened e.g. specimen transportation and analysis
		EVD transmission to communities in Uganda was prevented
		Cross-border surveillance teams were established
3.0	Improved cross-border collaboration	Cross-border surveillance teams were established
		Cross-border collaboration between Uganda and DRC improved
		Improvement in sharing of information between Uganda and DRC
4.0	Improved infection prevention and control (IPC)	IPC committees were established at health facilities
		Personal protective equipments (PPE)s were procured, e.g. coveralls, gloves
		Handwashing facilities were procured
5.0	Improved coordination for preparedness and response	Incident management system was adopted
		District task forces were operationalized in high-risk districts
		Response plans were developed at national and district levels
		Rapid response teams were formed
6.0	Community empowerment	Community sensitization was conducted
		Communities are more aware of VHFs and other diseases
		The community is more vigilant
		Information education communication (IEC) materials were distributed in communities
7.0	Supplies and logistical support	PPEs were procured
		Laboratory supplies were procured at national and sub-national levels
		Vehicles were procured to support the transportation of samples
		Ebola treatment units were established
		Isolation units were established
		Solar panels were installed in some health facilities

**Knowledge and skills gain:** many participants pointed out that health workers gained knowledge through training, which empowered them with essential skills to conduct outbreak investigations of viral hemorrhagic fevers and other outbreaks. Respondents at national and district levels pointed out that the health workers in health facilities feel more confident to respond to EVD outbreaks. “*There was a time when a bleeding patient sought for care in one of our health facilities and all health workers ran away and abandoned the patient, a team from national level had to come and assess the patient..*.” (respondent at district level). At the national level, many respondents cited training the national taskforce (NTF) in the incident management system, operation of the public health emergency center, leadership and partner coordination, operational planning as a critical achievement of the program. In contrast, district level respondents highlighted training the district task forces (DTF) as a significant achievement. Some of the respondents cited training of the village health teams (VHTs) on epidemics as a substantial achievement. The respondents reported that the VHT training was widescale and covered all villages in targeted districts. “*A community-based surveillance was conducted for all the VHTs in our district and because of this training, the VHTs are now more vigilant in reporting community alerts to health facilities..*.” (district level respondent). Simulation exercises were also mentioned as one of the critical activities that empowered health workers and other stakeholders with skills and knowledge on how to respond to EVD. “*We conducted a simulation exercise in Kasese District and immediately after this exercise, the EVD epidemic was imported into Uganda. The presentation of these cases was exactly similar to the scenario in the simulation exercise and this helped the frontline health workers to respond swiftly..*.” (national level respondent).

**Strengthening public health surveillance:** the respondents commonly cited improved case detection for EVD and other viral haemorrhagic fevers. Some respondents observed that the EVD preparedness and readiness program also strengthened case detection for other priority diseases such as polio, measles and cholera. The specific inputs and activities that supported improved case detection included the distribution of case definitions across health facilities, procurement of thermo-scanners and infra-red thermometers for Ebola screening and the deployment of screening teams at points of entry. “*At Mpondwe, a thermo-scanner was installed and this greatly improved the screening of travellers. Before this, a team of volunteers was trained and equipped with infra-red thermometers, infection prevention and control supplies*” national level respondent). “*...and community-based surveillance was enhanced through empowering VHTs to detect and report priority diseases which has helped us to detect priority diseases early..*.” (district level respondent).

**Improved cross-border collaboration:** under this thematic area, three main successes were attributed to the program and these included the establishment of cross-border surveillance teams, enhanced cross-border cooperation between Uganda and the DRC and improvement in sharing of information between Uganda and the DRC. The strong cross-border collaboration was highlighted as one of the major reasons why cross border transmission of the EVD epidemic to Uganda was limited to only a few cases despite the EVD epidemic epicentre being less than 100 km from the Uganda-DRC border. “*In June 2019, Ebola cases from DRC spilled into our district. However, before the suspected cases crossed into Uganda, our colleagues in DRC had alerted us of high-risk contacts who had escaped monitoring by surveillance officers. By the time these cases arrived at the health facility where they sought care, the health workers were already on high alert*” (district level respondent).

**Improved infection prevention and control (IPC):** the benefits reported most commonly under the IPC thematic area were establishing IPC committees at health facilities, provision of personal protective equipment (PPE) and procurement of handwashing facilities to enhance hygiene at health facilities and public places.

**Improved coordination for preparedness and response:** many respondents pointed out that the EVD preparedness and readiness program was associated with improvements in coordination for preparedness and response to disease outbreaks. The incident management system (IMS) 's adoption, operationalization of district task force (DTF) sub-committees and development of response plans at national and district levels were explicitly pointed out as the greatest benefits. “*For the first time, we adopted and strictly followed the incident management system. An incident commander was appointed to whom the heads of the different sub-committees reported daily*” (national level respondent). “*With support from the ministry of health and WHO, district sub-committees were formed and these included coordination, surveillance..*.” (district level respondent). “*We developed a stakeholders' matrix which streamlined coordination and resource mobilization and utilization*” (national level respondent).

**Community empowerment:** the main benefits elicited under this thematic area included community sensitization which made communities more aware of viral hemorrhagic fever (VHFs) and other diseases, increased community vigilance and distributed information education communication (IEC) materials in communities in targeted districts. “*Religious leaders, cultural leaders and opinion leaders were all involved in the implementation of activities which made it easy to deliver sensitization messages to communities*” (district level respondent). “*...and in addition to training of health workers, the VHTs and opinion leaders were also oriented on Ebola and this empowered them to participate in activities fully..*.” (district level respondent).

**Supplies and logistical support:** under this theme, procurement of personal protective equipments (PPEs), laboratory supplies, vehicles and solar panels at health facilities and establishment of Ebola treatment units (ETUs) and isolation units (IUs) were the commonest benefits mentioned by the respondents. “*Laboratory supplies including specimen transportation containers, triple packages and gloves were procured and delivered to the high-risk districts. This made it easier for frontline health workers to investigate suspected Ebola cases*” (national level respondent). “*Vehicles were procured to support transportation of samples from districts to the national reference laboratory*” (national level respondent).

**Factors that contributed to the success of the program:** in assessing the factors that contributed to the success of the EVD preparedness and readiness program, 92.6% (25/27) of respondents reported donor support and funding, 85.2% (23/27) reported adequate technical capacity, good coordination among stakeholders and political will and government commitment. Also, 81.5% (22/27) reported health workers' commitment and community involvement (Table 4). “*...in addition, we had a lot of support from WHO, United Nations International Children's Emergency Fund (UNICEF), Baylor and other partners and this streamlines district operations*” (district level respondent). “*The district political leadership was very supportive and was involved in planning and activity implementation. This made it easy to penetrate communities to provide EVD prevention messages..*.” (district respondent). “*Whenever it comes to implementing public health programs, the Uganda government is always very supportive. With physical security guaranteed for response teams, controlling epidemics is always easy*” (national level respondent)). “*Our health workers were very aware of the ongoing EVD epidemic in DRC. The fear of EVD alone made the health workers very committed to protecting the district from the Ebola threat*” (district level respondent).

**Table 4:** factors which contributed to the success of the program respondents in the EVD preparedness and readiness evaluation, Uganda 2020

Success factors	Number	Percent (n=27)
Donor support	25	92.6%
Adequate technical capacity	23	85.2%
Good coordination among the stakeholders	23	85.2%
Political will and government support	23	85.2%
Health workers' commitment	22	81.5%
Community involvement	22	81.5%
Other	5	18.5%

**Program's value for money:** a total of 25/27 (92.6%) respondents agreed that the EVD preparedness and readiness program demonstrated good value for money. Further assessment of whether the program demonstrated good value for money was conducted across the different outbreak response pillars. The majority of respondents agreed that there was good value for the money invested across the different response areas; coordination - 96.3% (26/27), surveillance and laboratory - 96.3% (26/27), case management and IPC - 92.3% (24/26), IPC - 90.9% (20/22), risk communication - 92.6% (25/27), psychosocial support - 76.9% (20/26) and logistics - 84.6% (22/26) (Table 5). Many respondents mentioned leveraging the capacities developed during EVD preparedness to respond to the COVID-19 pandemic in Uganda as the most significant example of good value for money: “*In many pillars of response, we are leveraging the EVD capacities for COVID-19 response. The rapid response teams at national and district levels are ready to respond even when COVID-19 is a new disease. Had it not been for EVD preparedness efforts, we would probably be struggling with COVID-19 amidst quarantine and lockdown*” (national level respondent). “*The knowledge we gained has been actually utilized beyond Ebola. In my district, we are actually applying EVD knowledge to do COVID-19 surveillance and screening*” (district level respondent).

**Table 5:** opinions of respondents about the program's value for money respondents in the EVD preparedness and readiness evaluation, Uganda 2020

Assessment of value for money	Agree		Disagree		Not sure		Total
	n	%	n	%	n	%	
Overall, there was value for money	25	92.6%	1	3.7%	3	3.7%	27
Coordination pillar	26	96.3%	1	3.8%	0	0%	27
Surveillance and laboratory pillar	26	96.3%	1	3.7%	0	0%	27
Case management and IPC pillar	24	92.3%	2	7.7%	0	0%	26
Risk communication pillar	25	92.6%	0	0%	2	7.4%	27
Psychosocial support pillar	20	76.9%	5	19.2%	1	3.8%	26
Logistics pillar	22	84.6%	2	7.7%	2	7.7%	26

Many respondents also cited knowledge gain as evidence for value for the funds invested during the implementation of the EVD preparedness and readiness program. Some respondents observed that rapid response teams were trained and always ready and confident to attend any disease outbreak. Other respondents observed that the EVD outbreak in Kasese district was rapidly contained because the response teams were well equipped with essential skills, knowledge and logistics. As a result, the teams could effectively detect and manage imported EVD cases and avert cases from transmitting EVD infection to the community and among health workers. “*...despite these challenges, the district and the Ministry of Health ably managed the EVD outbreak with no community transmission. This could not have been possible had it not been to EVD preparedness work and investments made*” (district level respondent). Other opinions elicited from the respondents about value for money included building capacities at national and district levels, availing supplies for outbreak response, improved community awareness, VHT empowerment and the establishment of ETU and IUs.

The lowest level of agreement about the program's value for money was observed in the psychosocial pillar, where 76.9% of respondents agreed that there was value for money (Table 5). Respondents cited a lack of psychologists and psychiatrists within the district structures, making it challenging to provide these services. One out of 27 (3.7%) respondents thought that the entire EVD preparedness program had no value for money. A few respondents disagreed that the investment demonstrated good value for money under the different response pillars. The highest level of disagreement observed was under the psychosocial pillar (19.2%; 5/26) (Table 5). Various opinions were obtained from the respondents who disagreed that there was good value for money under the program including spending funds on temporary structures, lack of adequate numbers of qualified teams to implement some activities and lack of response pillar specific drills. “*We did not have training specifically for psychosocial support and in cases of an outbreak, we only rely on regional and national levels to provide these services*” (district level respondent). “*The program's impact was low because most of the developed capacities were short-lived in nature. For example, the tents which were used to establish treatment centres and isolation units have worn out. The funds should have been used to build stronger and sustainable systems*” (national level respondent).

**Opinions about the availability of cost-effective alternative approaches:** when we assessed opinions about the availability of cost-effectiveness alternative interventions that could have delivered the same or similar results, 34.6% (9/26) of respondents believed there were cost-effective alternative interventions and 23.1% (6/26) believed there were no cost-effective alternative interventions. However, up to 42.3% (11/26) of respondents were unsure whether other, more cost-effective strategies were available (Table 2). Qualitative data interviews were conducted to understand whether more cost-effective alternative interventions could have delivered the same results. The five alternative interventions frequently mentioned included: i) conducting training using a mentorship approach; ii) online training approaches; iii) regular simulation exercises and drills; iv) integrating the EVD program within other public health programs; and v) supporting the government to build more permanent and sustainable structures. “*As a way of saving costs, health workers' training can be conducted using the online method or mentorships. This method can help avoid disruptions of health service delivery since health workers spend a lot of time travelling to attend different training courses..*.” (district level respondent). *...and because we have been experiencing EVD outbreaks, it's high time to consider integrating it within other routine services offered..*.” (district level respondent).

**Alignment of the program to the country's public health priorities:** a total of 22/26 (84.6%) reported that the program was well aligned with national public health priorities (Table 2). The most frequent explanation given by respondents was that EVD is one of Uganda's significant priority diseases. “*Ebola and other viral haemorrhagic fevers are part of the health problems in Uganda and therefore the program was well aligned to the country's priority needs*” (national level respondent). “*The program was well aligned with the national and district priorities. Uganda is one of the countries that gets EVD outbreaks and other public health emergencies. Many alerts of viral haemorrhagic fevers and other disease are now being reported*” (national level respondent). Some respondents noted that the availability of plans, guidelines and protocols ensured that the EVD preparedness and readiness program was well aligned with national priorities. “*The alignment to national priorities was super. The ministry of health developed a plan that divided the districts into three categories based on risk level: category I, II and III. The EVD preparedness program was implemented in districts with the highest risk (category I)..*.” (national level respondent). “*All district-level interventions were aligned to national health system plan for public health emergencies...and in addition, all districts developed preparedness and response plans based on national guidance*(national level respondent). Other justifications about the alignment of the EVD preparedness and readiness program with national priorities included: i) the activities were discussed and agreed upon by the ministry of health; ii) the program was implemented in line with IHR (2005) and global health security agenda (GHSA) [16] priorities; and iii) the program's contribution to the strengthening of capacities for responding to other priority diseases.

**Recommendations to improve EVD preparedness and readiness:** the recommendations made by respondents were grouped into five major themes, namely: i) to sustain capacity development; ii) to improve funding for preparedness and response activities; iii) to increase community involvement in preparedness and response; iv) to strengthening coordination; v) adoption of innovative practices for public health interventions; and vi) training more rapid response team members in districts (Table 6). Under capacity development, respondents emphasized the need for knowledge and skills improvement through regular refresher training, training of more case management teams, simulation exercises, drills and mentorships. The ways suggested to improve funding for response activities included establishing contingency funds at national and district levels and increasing the emergency response budget allocation. Regarding coordination, respondents indicated a need for better coordination among partners to avoid duplication of efforts and maximize resource utilization. “*We have had instances where you have three partners holding the same training within the same period with the same target. For this, the ministry of health should take the lead and coordinate implementing partners so that there is no duplication of efforts"*(district level respondent).

**Table 6:** suggestions to improve EVD preparedness and readiness, EVD preparedness and readiness evaluation, Uganda 2020

No	Theme	Main issues/achievements reported
1.0	Capacity development	Improve knowledge and skills for epidemic preparedness and response, e.g. simulation exercises and drills, train case management teams, refresher training, mentorships
		Establishment of border health points
		Pre-positioning supplies and logistics
		Transforming the established temporary Ebola treatment/isolation centres into permanent structures
2.0	Improved funding for preparedness and response activities	Establish a national level contingency fund for emergency response
		Establish a district-level contingency fund for emergency response
		Increase the emergency response budget
3.0	Community involvement in epidemic preparedness and response	Involve VHTs
		Involving local council authorities
		Involving opinion leaders
4.0	Strengthening coordination	Improve partner coordination
		Strengthen district task forces
5.0	Adoption of innovative public health interventions	Integrating EVD response into routine care
		Adoption of the IDSR-3 framework
		Integrating preparedness and response to existing health service delivery mechanisms
6.0	Strengthening district response structures	Adoption of a district-led response
		Districts should be more involved in planning
		Training more district rapid response team members

Some participants suggested that the ministry of health adopt innovative interventions such as integrating EVD response into routine care, the integrated disease surveillance and response (IDSR) version 3 framework and integrating preparedness and response into existing health service delivery mechanisms. There was a suggestion to strengthen the district response structures further to control disease outbreaks and public health emergencies effectively. Respondents suggested that more district rapid response teams be trained and that the districts should lead all response activities. “*Most of the interventions start from the centre to the district. The teams are deployed to the districts by the ministry of health and partners. This approach is costly, not sustainable and hardly allows the districts to develop context-specific solutions. Rather than having this approach, partners can consider supporting and building local capacity and provide technical oversight rather than actually doing the implementation themselves..*.” (district level respondent).

## Discussion

Between May and September 2020, we conducted an independent evaluation of the WHO and partner supported EVD preparedness and readiness program in Uganda to document any achievements and determine if they represented good value for the funds and resources invested. We explored if there were more cost-effective or sustainable alternative approaches that could have delivered similar results. We assessed whether the investments into EVD preparedness and readiness were aligned with Uganda's priority needs to improve health system security in line with their IHR (2005) commitments. We also documented what other factors contributed to the success or failure of the EVD preparedness and readiness program interventions. We found that the EVD preparedness and readiness program was a success. This was attributed mainly to donor support, the ministry of health's technical capacity, good coordination, government support and community involvement. The resources invested in the program represented good value for the funds received and the activities were well aligned to the public health priorities. However, there were mixed (i.e. equal numbers for and against) responses on whether or not there were more cost-effective or more sustainable alternatives that could have produced similar results in Uganda.

These findings are significant because the capacities that were developed under EVD preparedness and readiness have been transitioned to the ongoing COVID-19 pandemic response through the implementation of IDSR [17-19]. The EVD preparedness and readiness program in Uganda was part of the UN and development partners' widescale interventions to avert the spread of EVD from the DRC to neighbouring countries to maintain regional and global health security. We found that the program's interventions significantly improved public health surveillance and response, which is critical for EVD preparedness and response. Uganda was one of the first countries to implement IDSR, which is the basis of public health surveillance and response and IHR (2005) in the country. IDSR began in Uganda in 2000 and has been used to investigate and respond to viral hemorrhagic fever outbreaks and other outbreaks since. Adopting and implementing the revised IDSR framework (version 3, [20]) needs to be accelerated in Uganda. From 2012 to 2017, WHO and country partners supported Uganda to revitalize IDSR and the capacities that were developed were available for the EVD preparedness and readiness program. The funds and logistical support that were available for the EVD preparedness and readiness program also contributed to the overall strengthening of IDSR. The results of the IDSR revitalization project continued into the EVD preparedness and response program [18,19,21].

A lack of sustained funding for IDSR implementation was a shortcoming identified by Lukwago *et al*. [22] and highlights the need for continued investment in all elements of IDSR to maintain adequate performance and thus health security. A sustainable funding source needs to be identified to sustain IDSR performance in Uganda and the other WHO-AFRO member states. Public health surveillance and laboratory systems are essential for preventing, detecting and controlling public health emergencies. In addition to capacity development through training, the EVD preparedness and readiness program provided supplies and tools to enable the trained personnel to perform the surveillance and response tasks. These tools included PPE, laboratory supplies, vehicles and solar panels at health facilities and establishment of ETUs and IUs; this addresses a shortage described in the evaluation of the Uganda IDSR revitalization [18]. Converting the temporary health posts into permanent structures at the border with the DRC (and other countries) will enable the necessary items and personnel to be positioned on-site for prompt detection and response to public health emergencies and using permanent health posts as a hub for supporting EVD vaccination. Cold chain infrastructure could be positioned at points of entry (POEs) to support EVD and other vaccination programs.

The EVD outbreak in West Africa showed that Ebola epidemics quickly spread because of substantial weaknesses in surveillance and reporting systems which led to delayed outbreak detection and global response [23]. As a result, the epidemic spread from Guinea to other neighbouring countries and eventually led to over 11,000 deaths [24]. The EVD preparedness and readiness program strengthened the technical capacity within the ministry of health and donor support contributed to technical support, capacity building and supplies and logistics. Community involvement was one of the significant factors contributing to the success of the EVD preparedness and readiness program. The community engagement approach leveraged relevant stakeholders at national, district and community levels. At the national level, the ministry of health, WHO and other UN agencies and implementing partners led the planning, design and coordination of interventions and provided technical support. The ministry of health and implementing partners conducted intensive community engagement to ensure that the community-supported and fully participated in delivering critical preparedness interventions. Intensive community sensitization through media and places of worship was conducted and religious leaders, cultural leaders, opinion leaders and village health teams (VHTs) were oriented on Ebola detection, reporting, prevention and control. Early community engagement in Uganda was critical because lessons learned from the DRC had already shown that there was a disruption of response efforts due to community resistance and distrust of responders [25-27]. Community engagement has been identified as a lesson that has been learned from the EVD response that should be transferred to the COVID-19 response [28].

Innovative training approaches that leverage digital health technologies, cellular networks and online platforms are likely to become more relevant as cellular services improve in Africa. In several cases, the frontline health workers who respond to EVD and other outbreaks are in remote areas that may be hard to reach, yet they need regular, refresher training. The restrictions and curfews during COVID-19 have magnified this challenge. Sierra Leone and other countries have demonstrated case study examples for mobile-based training and support training for EVD (MOTS) [29]. Uganda has used a short message service (SMS) based system called “mtrac” for IDSR and health management information system reporting and training. Leveraging cellular networks is likely to be a cost-effective option for training, reporting and supervision [30].

Our evaluation had some limitations. The main limitation is that we conducted it during the COVID-19 pandemic with curfews and limited movement, which could have adversely affected the response rate and the type of information we could obtain. The other limitations are: first, the evaluation participants could have views that were influenced by their role in the EVD preparedness and readiness program, which have led to a natural bias to focus on program successes. However, the evaluation team tried to tease out the program weaknesses during interviews. Secondly, EVD preparedness activities were mainly implemented in 20 high-risk districts in Uganda, but due to logistical reasons and the COVID-19 pandemic, the evaluation could not be done in all districts. Therefore, it is possible that the districts that were not surveyed could have had different results from the ones that were studied. Thirdly, due to COVID-19 restrictions, we were limited in the number of respondents we could access and it is possible that the views of the health workers we could not access would be different from those that we could access, especially some frontline health workers in the periphery. However, it should be noted some of the members of the district health team are frontline health workers appointed to perform extra functions, e.g. district surveillance focal persons and laboratory surveillance focal persons.

## Conclusion

We conclude that the EVD preparedness and readiness program in Uganda developed capacities that played an essential role in preventing cross border spread of EVD from the affected provinces in the DRC and enabled rapid containment of two importation events in 2019. These capacities are now being used to detect and respond to the COVID-19 pandemic. We made the following recommendations after the evaluation. First, to strengthen public health surveillance by adopting the revised IDSR version 3 framework [20] in Uganda. Second, to establish permanent health posts at the borders, particularly the border with the DRC. Thirdly, to establish an electronic platform for village health teams to report community alerts and events timelier. Fourth, to improve supplies and logistics by pre-positioning PPEs in high-risk districts to enable rapid response during emergencies and convert the temporary Ebola treatment units and isolation centres into permanent structures. Fifth, to improve training activities by training more case management teams, conducting regular simulation exercises and considering increasing the use of more cost-effective strategies such as online training and mentorships and periodic refresher training for district-level rapid response teams. Finally, to provide consistent funding for preparedness and response activities by establishing a contingency fund at national and sub-national levels to enable an immediate response to public health emergencies.

**Funding:** funding for the study was provided from the WHO emergencies and preparedness programme within the WHO regional office for Africa.

### What is known about this topic

- The Democratic Republic of Congo (DRC) has experienced recurrent Ebola virus disease (EVD) outbreaks for several years;
- There is a continuing risk of spread of EVD from DRC to surrounding countries during the recurrent EVD outbreaks;
- Countries neighboring DRC need to develop strategies to mitigate EVD spread from DRC.

### What this study adds

- Uganda which neighbors DRC was supported by WHO and partners to improve EVD preparedness and response;
- We evaluated and found that the EVD preparedness and readiness program was a success, and this was attributed mainly to donor support, the ministry of health's technical capacity, good coordination, government support and community involvement;
- Several capacities were developed to prevent and respond to EVD importations in Uganda which are now being used to respond to the COVID-19 pandemic.
